# Endothelial cell provenance: an unclear role in transplant medicine

**DOI:** 10.3389/frtra.2023.1130941

**Published:** 2023-04-28

**Authors:** Autumn Pace, Marie E. Steiner, Gregory M. Vercellotti, Arif Somani

**Affiliations:** ^1^ University of Minnesota Medical School, Minneapolis, MN, United States; ^2^ Department of Pediatrics, Division of Hematology/Oncology, University of Minnesota Medical School, Minneapolis, MN, United States; ^3^ Department of Pediatrics, Division of Critical Care Medicine, University of Minnesota Medical School, Minneapolis, MN, United States; ^4^ Department of Medicine, Division of Hematology, Oncology, and Transplantation, University of Minnesota Medical School, Minneapolis, MN, United States

**Keywords:** endothelial cell provenance, endothelial chimerism, cellular transplantation, organ transplantation, hematopoietic stem cell transplantation

## Abstract

An understanding of the interplay between both donor endothelial progenitors and the recipient endothelium (in the case of hematopoietic cell transplant) and recipient endothelial provenance upon the established donor endothelium (in the case of solid organ transplant) is unknown. It is postulated that this interplay and consequences of purported dual endothelial populations may be a component of the post-transplant disease process and contribute to complications of engraftment or rejection. To address this potential confounding and often overlooked arena of vascular biology, a directed brief overview primarily focused on literature presented over the last decade is presented herein.

## 1. Introduction

Endothelial cells comprise a physical and functional interface between blood and tissues, and in the context of transplant medicine, between self and non-self. Beyond their role in metabolic hemostasis, endothelia provide biological linkages in the dynamic regulation of vascular tone, permeability, coagulation, and inflammation (1). Endothelial cells express Class 1 and Class II MHC antigens, ABO antigens and a variety of surface molecules in response to ischemia/reperfusion physiology, cytokine exposure and cell injury pathways. Human endothelial cell can act as antigen presenting cells to T cells *via* LFA3/CD2, CD45 and allo antibody responses leading to organ rejection. Pre-formed endothelial antibodies in recipients can further fuel this process. The endothelium is exposed to inflammatory cytokines, alloreactive lymphocytes, activated neutrophils, donor-specific antibodies, procoagulant proteases and complement fragments. This leads to further endothelial cell activation and potentially organ rejection or graft vs. host disease (2–4). Recipient endothelial cells that repave the vasculature with HLA and ABO compatible surfaces may be a homeostatic attempt to attenuate this inflammatory process. Hence, an understanding of the provenance of the endothelial cell may yield clinical implication in terms of graft function and survival.

The transplant population has grown in recent years with 22,013 hematopoietic stem cell transplants performed in the United States in 2020 including both pediatric and adult cases (5). Similarly, 33,309 solid organ transplants were completed in 2020 according to the Organ Procurement and Transplantation Network data (6). Both cellular and solid organ transplants face potential compromised graft and host viability from required immunosuppressive medications, resultant infections and both acute and chronic rejection (6). While this is clearly documented in the literature, an understanding of the interplay between both donor endothelial progenitors and the recipient endothelium (in the case of cellular transplant) and recipient endothelial ontology upon the established donor endothelium (in the case of solid organ transplant) is unknown. It is postulated that this interplay and consequences of purported dual endothelial populations (i.e., of donor and recipient origin) may be a component of the post-transplant disease process. To address this potential confounding and often overlooked arena of vascular biology, a directed brief overview primarily focused on literature presented over the last decade is presented herein. Moreover, given that both cellular and solid organ transplant present complementary yet inverse donor and host endothelial interactions, both processes are subsequently alluded to.

**Figure 1:**
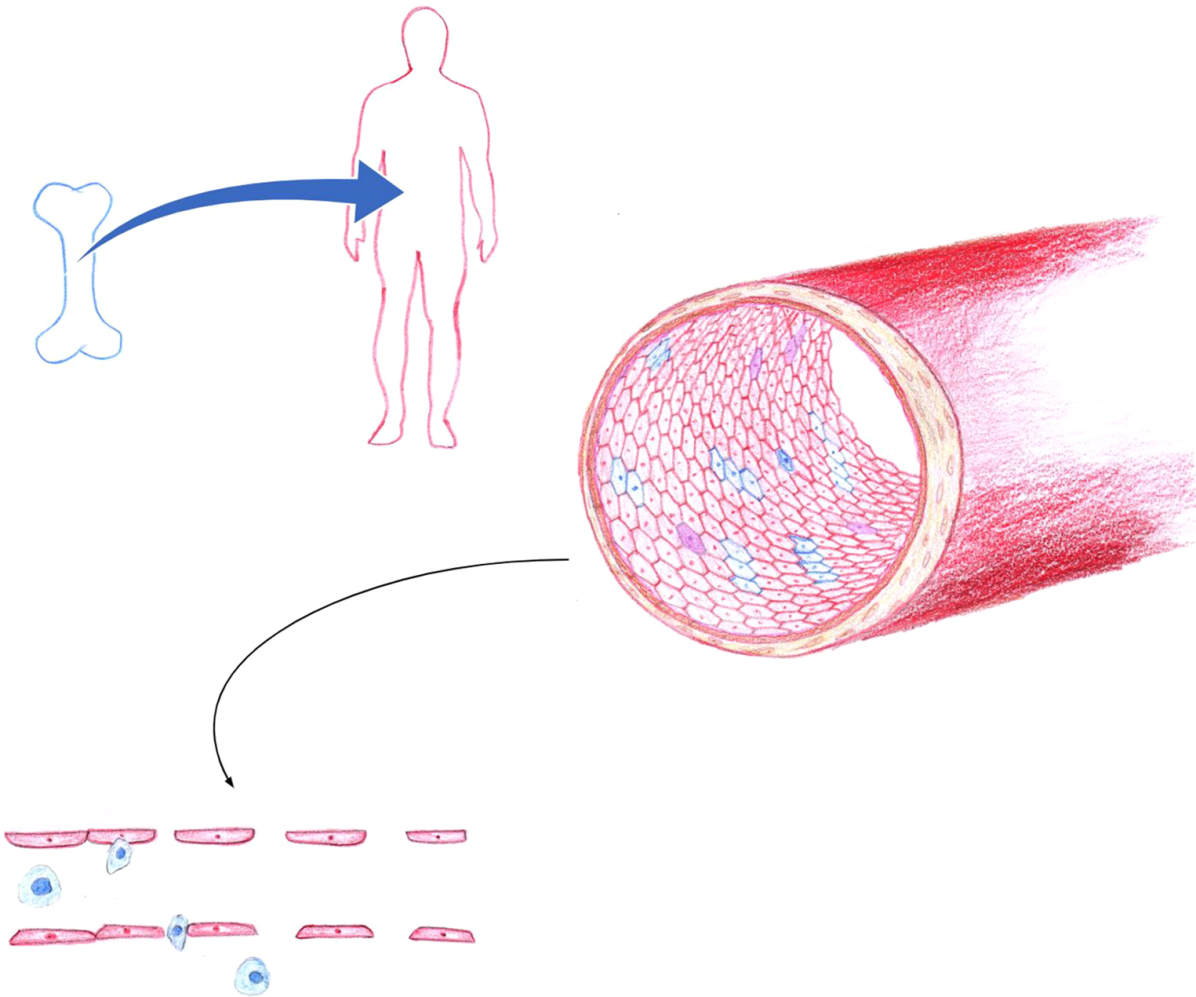
In hematopoietic stem cell transplantation, endothelial chimerism is a result of the process by which recipient endothelium is gradually repopulated by endothelial progenitor cells of donor bone marrow origin. Blue, indicates of donor origin. Red, indicates of recipient origin. Purple represents monocyte to endothelial transformation or fusion cell phenomena. Endothelial dysfunction is exacerbated by HLA and ABO disparity, along with loss of tight junction integrity, overexpression of adhesion molecules that promote leukocyte recruitment and transmigration across the endothelium. The resultant endotheliopathy contributes to the pathogenesis of graft versus host disease, sinusoidal obstruction syndrome, capillary leak, transplant associated thrombotic microangiopathy, and idiopathic pneumonia syndrome. (Figure by S. Somani).

**Figure 2:**
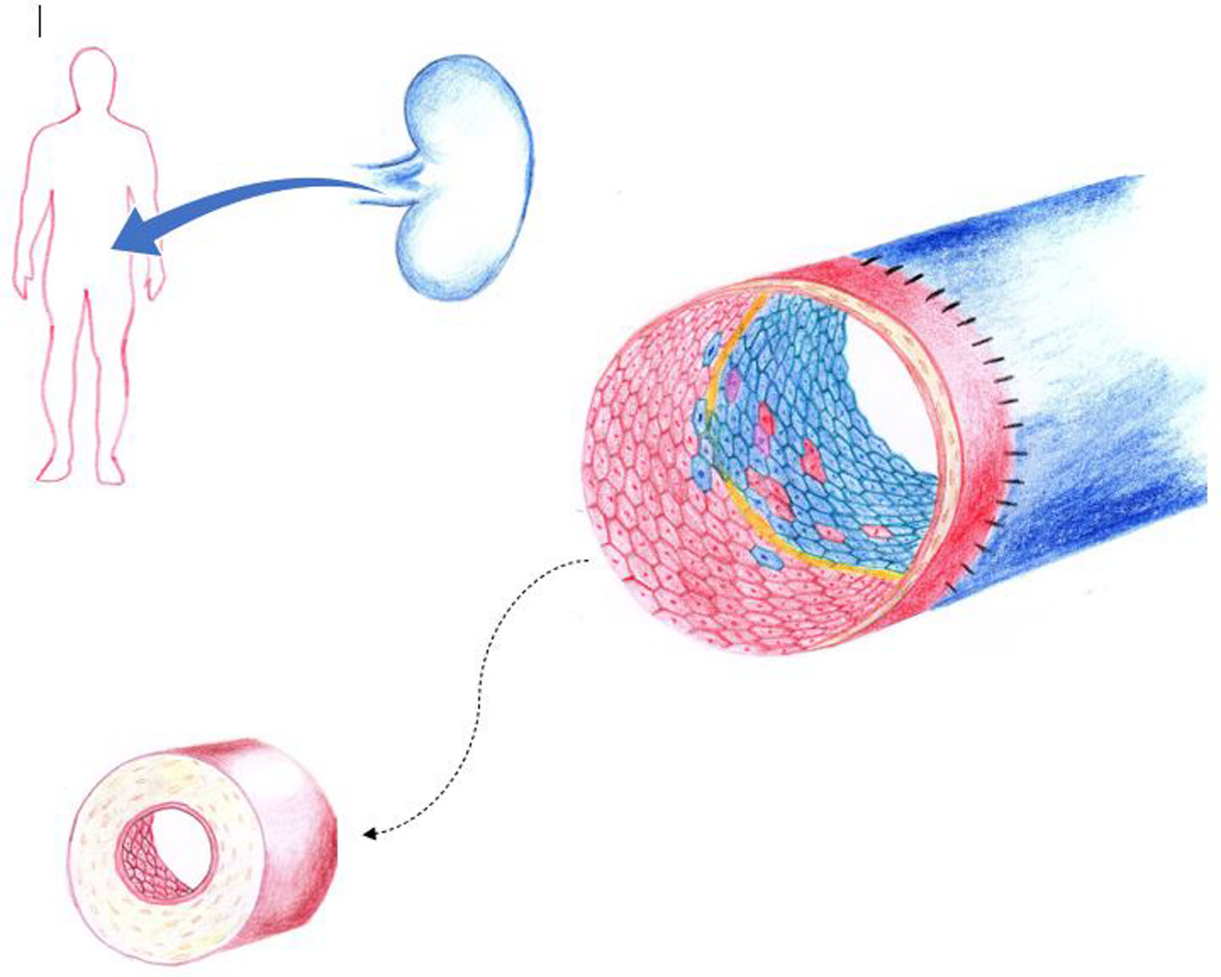
In solid organ transplantation, organ derived mature senescent donor endothelial cells are transplanted with the organ graft. Here, reverse endothelial chimerism occurs as recipient-derived cells replace the donor-derived endothelial cells within the vasculature of the grafted organ. At the zone of vascular anastomosis, exposed basement membrane (yellow) may also be reendothelized by donor graft endothelial cells (facilitated by cell-to-cell contact expansion). Blue, indicates of donor origin. Red, indicates of recipient origin. Purple represents monocyte to endothelial transformation or fusion cell phenomena. Chronic alloimmune injury leads to intimal thickening, accumulation of extracellular matrix, smooth muscle cell proliferation with resultant luminal narrowing. Here an indolent host versus graft reaction results in transplant vasculopathy that is associated with long-term organ loss. (Figure by S. Somani).

Given that transplant rejection is a common occurrence, there have been many studies aimed at improving the understanding of this pathophysiological process. One of these hypothesized mechanisms may be related to the concept of endothelial chimerism at the organ level, whereby donor and host endothelial cell populations both line the vasculature. Endothelial chimerism varies depending on the type of transplant which is being discussed. In HSCT patients, endothelial chimerism is a result of the process by which recipient endothelium is gradually repopulated by immature donor-derived cells of ontological donor bone marrow providence (7) (Figure 1). In solid organ transplantation, organ derived mature senescent donor endothelial cells are transplanted with the organ graft. For this reason, it is commonly referred to as reverse endothelial chimerism, which is defined as recipient-derived cells replacing the donor-derived endothelial cells within the vasculature of the grafted organ (8) (Figure 2). Endothelial or reverse endothelial chimerism may be assessed in a variety of ways, using fluorescence *in situ* hybridization (FISH), immunohistochemistry (IHC), or flow cytometry to evaluate sex-mismatched transplants, ABO-incompatible transplants, and/or unique genetic markers (7, 9–11). Age-associated vascular changes may further affect the endothelial chimerism occurring after transplantation. With aging, vessel density and pericyte numbers decline significantly in tissues displaying lower remodeling capacity (such as the kidney, muscle, and spleen) vs. tissues with a greater regenerative potential (such as the gut, skin, uterus, and the human liver). Secondly, at the cellular level accumulation of reactive oxygen species, low grade inflammation, mitochondrial dysfunction, and even pericyte to fibroblast differentiation that occur with aging may be compounded and triggered by vascular injury and could be expected to develop from chemotherapy, radiation, infection, or surgical manipulation occurring in transplant settings (12). Likewise, the aged bone marrow has limited lymphatic endothelial cell expansion ability, diminished cellular cross-talk capacity, and attenuated hematopoietic stem cell (including EPCs) regeneration (13). The age-associated tissue-specific molecular changes could thus variably repopulate the endothelium following transplant with unknown consequences but has not been specifically studied.

In 1965, Medawar hypothesized that replacement of donor vascular endothelium by host endothelium may lead to increased survival of the graft (presumably by allowing for preservation of microvascular architecture and function that would otherwise be obliterated by immune mediated acute or indolent rejection) (14). At that time, studies had predicted the site of graft rejection was against the donor endothelium (15–18). Complementary to this concept, Calne suggested that early reverse endothelial chimerism would protect the donor endothelium from graft rejection and improve viability of graft acceptance (18). These controversial topics are still widely debated and continue to be an active area of investigation in transplant medicine.

## 2. Methods

A literature search using the keywords “endothelial” “chimerism” and “transplant” was conducted within the PubMed database. The results were filtered to only include publications which were published between 2010 and 2020 to summarize current knowledge. This resulted in 43 abstracts, which were reviewed to determine possible pertinent papers.

**Figure 3:**
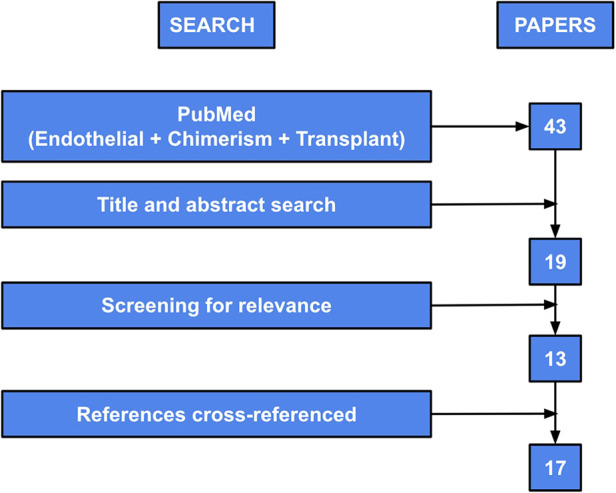
Diagram of search methods used to perform systematic review. “PubMed” refers to the initial keyword search within the PubMed database. “Title and abstract search” refers to review of titles and abstracts and exclusion per exclusion criteria noted above. “Full article search” refers to full paper review and exclusion at that point per exclusion criteria noted above. “Screening for relevance” refers to assessment of the publications’ relevance to this review. “References cross-referenced” refers to review of the references from the original included papers and the addition of papers if they satisfied inclusion and exclusion criteria. Figure adapted from Bolado and Landin (19).

Abstracts were excluded at this point if the entire paper was not available or printed in English, if it was a duplicate article, or if there was duplicate data published which had been included in a previous paper. Of these 43 abstracts, 19 papers were selected for further screening for relevance to this review. Of the 19 papers that were included, 13 of these proved to be pertinent and included specific information related to this review. Manuscripts were excluded if they mentioned chimerism of various types of cells after transplant but did not specifically address or discuss endothelial chimerism. References of the final 13 manuscripts were cross-referenced and an additional 4 papers were added for further references (Figure 3).

## 3. Results

### 3.1. Endothelial chimerism after bone marrow transplant in animal models

Multiple studies have readily established that bone marrow-derived cells are the primary ontologic progenitors of mature endothelial cells, however the terminology, surface marker definition, quantity and doubling potential of donor derived endothelial cells is debated and varies between different studies (20). Within the past 10 years, two animal studies have been published that investigated endothelial chimerism after bone marrow transplant (10, 21). However, neither of these studies investigated any association with graft vs. host disease or transplant rejection.

Bonfim-Silva et al. demonstrated in a mouse model that endothelial chimerism happens frequently within the bone marrow as early as 30 days after bone marrow transplant (10). Green fluorescent protein (GFP) positive donor cells in GFP negative mice showed significantly more endothelial cells derived from the transplanted GFP + bone marrow than native cells from the GFP- recipients (39.58 ± 10.66% vs. 2.75 ± 0.9%, *p* = 0.04). Bone marrow derived cells (BMDC) are also recruited into the melanoma tumor microenvironment and contribute to vascular development. The GFP + bone marrow transplanted GFP- mice were found to have 11.5 ± 6.85% of GFP + cells present as CD31 + endothelial cells by flow cytometry. Additionally, these CD31+ GFP + endothelial cells were localized to blood vessels supplying the melanoma tumor microenvironment (10). While this study confirms that bone marrow derived cells can contribute to the bone marrow environment and tumor environment, the study does not evaluate or correlate its findings with clinical significance. Further, the tumor microenvironment is metabolically active with likely novel angiogenesis that may limit inferences into the more quiescent or senescent vascular beds.

A second animal study published within the past 10 years demonstrated that endothelial chimerism happens diffusely throughout multiple different organ systems. BXSB mice were transplanted with GFP + unfractionated bone marrow cells or *ex vivo* expanded mesenchymal stem cells delivered by intravenous injection (21). Organs that demonstrated endothelial chimerism at 62 weeks after injection included the liver sinusoids, brain choroid plexus and the endothelium of adipose, lung, and kidney tissue. GFP + chimeric endothelial cells were also found in the capillaries of the gut, skin, and striated muscle, but not within capillaries of the pancreas or brain parenchyma. While endothelial cells derived from transplanted unfractionated bone marrow was demonstrated in various organs in this study, the frequency at which these transplanted cells were found was not addressed. The authors speculated that the multisystem engraftment of endothelial cells following intravenous progenitor cell infusion coupled with immune modulation of the host and organ-specific factors might contribute to disease control through endothelial cell chimerism (21).

### 3.2. Bone marrow transplant in humans

Following human hematopoietic stem cell transplantation (HSCT), donor stem cells migrate into numerous tissues where they proliferate and differentiate, creating varying degrees of chimerism between recipient and donor cells. Pulmonary chimerism involving bronchial and alveolar epithelium and endothelium, including Type II pneumocytes, has been described in association with various lung injuries (22). In a recent study, Hijiya et al. studied pulmonary endothelial chimerism in patients who had previously received an ABO-incompatible hematopoietic stem cell transplant. Immunohistochemical staining to ABO antigens was used to determine the percentage of vessels expressing donor antigens on the pulmonary endothelium. Of the 16 samples which were analyzed, 7 of the samples came from explanted lungs in patients who had required pulmonary transplants for severe chronic pulmonary graft-vs. host disease (GVHD). The other 9 samples were obtained from autopsy samples with 6 of these 9 autopsy samples posthumously diagnosed with chronic pulmonary GVHD. Of the overall 13 samples which were diagnosed with pulmonary GVHD, all of them showed pulmonary endothelial chimerism. The frequency of donor group antigens on vessel endothelium ranged widely from 0.1 to 17.5% in these patients with pulmonary GVHD but no endothelial ABO chimerism was observed in the 3 samples from patients unaffected by GVHD (9.28% ± 6.59 vs. 0 ± 0, *p* < 0.001). There was also a positive correlation between percentage of chimeric vessels and recipient age at transplant (*r* = 0.85, *p* = 0.02), which may co-correlate with development of GVHD. A literature review included in this study tabulated 20 of 28 patients reported with endothelial chimerism and 5 of 11 with epithelial chimerism with different pathologies, including diffuse alveolar hemorrhage, bronchiolitis obliterans and “chronic inflammation” (22). Of note, transplant toxicities such as thrombotic microangiopathy are likely due to multi-factorial insults, but endothelial chimerism has not been clearly implicated as a pathogenic mechanism (23).

Skin GVHD has been associated with endothelial chimerism as well (22). Two cases reported by Kaffenberger et al. also demonstrated endothelial cell chimerism but within GVHD-associated angiomatosis (GVHD-AA) diagnosed at 46- and 30-months post-transplant respectively (24). The frequency or percentage of chimeric endothelial cells was not documented in either of these cases.

Tran et al. described endothelial chimerism in salivary glands after stem cell transplantation. Five females who transplanted from male donors who underwent salivary gland biopsy had scattered Y-positive cells in acini, ducts, stroma, and endothelial cells of their salivary glands (mean 1.01%) from 13 to 201 months following transplant. Four had GVHD (liver, skin, oral and/or cryptogenic organizing pneumonia) (25).

Mueller's series of endothelial chimerism included 52 HSCT patients who underwent a combination of 22 normal skin biopsies, 12 GVHD skin biopsies, 4 tumor biopsies, and 5 autopsies variably sampling heart, liver, skin, and marrow following HSCT (7). Analysis *via* ABO immunohistochemistry, XY fluorescence or short tandem repeat analysis of laser captured endothelial cells failed to show physiologic endothelial turnover resulting in donor endothelial chimerism. Endothelial cell chimerism was detected at low levels (0.9% and 3.3%) in skin biopsies from only two patients with chronic GVHD. Tumor tissues showed 1.2% and 2.5% of donor derived endothelial cells in two patients. The authors concluded that “endothelial cell replacement by bone marrow derived donor cells… is a rare event” and “does not represent a major repair mechanism”. However, they did not sample lung tissue in their patients (7).

Thus, the mechanisms by which circulating donor stem cells may populate vascular endothelial surfaces remain unclear. Prior injury or inflammation appears to be a precipitating factor and the circulating stem cells may contribute to a healing effect of regional or tissue-specific chimerism. Whether this chimerism is beneficial and can/should be facilitated in early stages of injury to mitigate severe adverse transplant-related toxicities, particularly in the lung, remains to be studied.

### 3.3. Solid organ transplant in animals

Three studies were conducted recently which investigated endothelial chimerism within solid organ transplants in animal models. In this situation, (reverse) endothelial chimerism is defined as having recipient-derived endothelial cells replace the donor-derived endothelial cells or co-populate within the vasculature of the grafted organ (8).

While Chen et al. primarily focused their study on pancreatic islet transplants, they made some comparisons to mouse models of heart transplants to evaluate how donor specific antibodies can lead to solid organ transplant failure (26). Syngeneic and allogeneic heart transplants were evaluated in a mouse model, which was injected with either donor specific antibodies or placebo (HB13 monoclonal antibody vs. phosphate buffered saline). At 30 days after cardiac transplantation, the transplant was harvested for histological evaluation. Cardiac transplants which were exposed to donor specific antibodies showed evidence of humoral rejection (as documented by complement activation, leukocyte infiltration, and destructive ultrastructural endothelial changes noted on staining and electron microscopy) while the hearts that were exposed to phosphate buffered saline did not. Additionally, the transplanted hearts were assessed using flow cytometry at 4 weeks post-transplantation and the endothelial cells were deemed to be of donor origin (although not quantified). In contrast, a progressive replacement of donor endothelial cells by recipient endothelial cells was observed over a six-week period in their pancreatic islet cell aggregate transplanted into the renal subcapsular area. Acknowledging that in solid organ transplant, immediate viability depends on establishing perfusion by surgical connection of prominent vessels (as in their cardiac model) vs. angiogenesis and diffusion capacity in cellular aggregate transplant (as in the their subcapsular islet cell model), they postulate that reverse endothelial chimerism and the diffusion restriction of large proteins (complement activators and donor specific antibodies) is protective against humoral mediated rejection in the latter situation, which is clearly not afforded in their cardiac transplant model (26).

Interestingly and in contrast, Onuta et al. found a positive association between the frequency of host-endothelial chimerism and the frequency of transplant vasculopathy (27). In their experiments, MHC-incompatible transplants were performed between various strains of rats, specifically Lewis and Brown Norway. After one and two weeks of MHC-incompatible aortic transplantation, the host-endothelial chimerism was assessed histologically. In the BN-to-Lew transplants, 2%–3% of endothelial cells were host derived; while in the Lew-to-BN transplants, 37% and 27% of endothelial cells were host derived at the respective one- and two-week time point post-transplant. This increased host-derived endothelial cell chimerism may be reflective of an injured intimal layer on the transplanted aortic graft and was correlated with a more pronounced profibrotic state and transplant vasculopathy noted over 4 to 8 weeks. Lew-to-BN grafts also had earlier, and more aggressive acute vascular rejection compared to BN-to-Lew allografts, which may be influenced by underlying non-MHC-immunologic determinants, intrinsic neointimal smooth muscle cell proliferative capacity and availability of host-derived fibrocytes. However, this is correlation, not causation, and the timeline and details regarding level of vascular rejection were not discussed within the study (27).

Schirutschke et al. attempted to quantify incorporation of nonrenal host endothelial cells (defined by double staining for RECA-1 and *hPAP*) in R26-*hPAP* transgenic Fischer F-344 rats (with confirmed hPAP positivity of all bone marrow cells) who received Fischer F-344 wild type rat kidney grafts (28). They used both an acute and reversible endothelial cell-specific nephritis model (GEN model with loss of 85% of the glomerular endothelial cells and a loss of 69% of the peritubular endothelium at day three post renal injury) and a complex, chronic progressive model of kidney endothelial injury (5/6 nephrectomy model with noted endothelial rarefaction of 23% in the glomeruli and 49% in the peritubular capillaries after 14-week post injury). Both models demonstrated infiltration of *hPAP +* cells (thought to be macrophages or inflammatory cells); however, limited incorporation of host endothelium was noted at both the glomerular (0.25% at GEN week 4 and 0.05% at 5/6 Nx week 14) and the peritubular level (0.1% at GEN week 4 and 0.86% at 5/6 Nx week 14).They conclude that independent of acute vs. chronic or healing vs. progressive disease outcome, actual recipient derived incorporated endothelium is a rare event and that endothelial regeneration likely originated primarily from intrinsic kidney cells in their syngeneic transplant model (28).

The syngeneic animal model does necessarily limit our inferences for most human transplantation situations.

### 3.4. Solid organ transplant in humans

An additional three articles have been published within our search time frame (2010–2020), further supplemented by a 2010 paper (29) and 2013 synopsis article (19) that investigated reverse endothelial chimerism of solid organ transplants within human patients and its association with transplanted organ rejection.

Tanabe et al. 2011 evaluated the rate of endothelial chimerism expression of blood type A or B antigens in the transplanted kidneys of 6 patients who had received ABO-*in*compatible kidney transplants over the 10 years post-transplant (10). In general, the expression of blood-type A or B antigen (on identified CD34 positive capillaries) decreased as the duration from transplant increased. Expression of blood-type A or B antigen decreased to 91.8%, 85.8%, 64.1%, and 57.6% in the respective first three months, five years, ten years, and greater than ten years post-ABO-incompatible kidney transplantation. In comparison to a control group of ABO-*compatible* transplant recipients, no change in blood-type A or B antigen expression was seen after transplant with 99.8% of vessel endothelium expressing the expected blood-type antigen more than 10 years after an ABO-compatible renal transplant. While (antigenic, not necessarily cellular) endothelial chimerism in the long-term period post-ABO-incompatible renal transplant was demonstrated here, it could not be associated with either graft accommodation *(i.e., resistance to humoral rejection despite the presence of antibodies against the donor endothelium)* or antibody-mediated rejection. Only one of the 6 patients was diagnosed with chronic antibody mediated rejection, which occurred about 7 years after ABO-incompatible transplant, however the rate of this patient's endothelial chimerism was similar compared to the remainder of the 5 patients. Moreover, similar graft and patient survival rates between ABO-incompatible and compatible kidney transplants are likely due to the efficacy of post-transplant immunosuppression regimens clouding inferences at the endothelial level. However, Tanabe et al. 2012 suggested that patients with acute or chronic antibody mediated rejection had a higher incidence of chimerism (7/9 patients), leading to poor graft survival (8). Hence, it is still unclear whether replacement chimerism may allow for graft adaption (whereby donor endothelial cells repopulate the donor's organ vessel walls) or are involved in graft compromise.

Varga et al. also evaluated the frequency of endothelial chimerism in sex-mismatched kidney allograft recipients (identifying XX or XY chromosomes *via* FISH or CISH) and its relationship to signs of rejection (9). 16 patients were evaluated 1–12 years duration after a sex-mismatched renal transplant. Endothelial chimerism was not noted in any of the 4 female recipients, however endothelial chimerism was noted in lymphatic vessels in 25% (3/12) of male recipients and in capillary vessels in 17% (2/12) of male recipients. In all the grafts which showed endothelial chimerism, tubular cell chimerism was also noted, so there were no grafts with isolated endothelial chimerism. In the 5 patients with demonstrated endothelial and tubular cell chimerism, 3 of these patients also had acute T-cell rejection, however this association was not statistically evaluated nor associated with antibody mediated rejection (9).

Ferlicot et al. evaluated the frequency of chimerism in sex-mismatched renal transplants using FISH (for the Y chromosome) and IHC (for endothelial marker CD31) in 33 renal biopsies from 22 male recipients who had received female kidney transplants (29). Endothelial cell chimerism was present in 67% of patients with a mean percentage of 61.8% chimeric glomeruli or a mean number of 3.53 chimeric cells per glomerular section. They did find endothelial chimerism was associated with a prior (but not necessarily acute current) episode of acute T-cell mediated rejection (*p* = 0.02). Moreover, having had higher grade II/III acute-T-cell mediated rejection appeared correlated to a greater number of chimeric cells per glomerular section compared to prior grade I rejection in these patients (29). This may support the contention that donor graft endothelium is replaced after rejection associated vascular injury.

Bolado and Landin published a review article evaluating a total of 33 articles published between 1972 and 2012 on the frequency of reverse endothelial chimerism in solid graft recipients of cardiac, kidney, liver, and lung transplants (19). The incidence of reverse chimerism was respectively 50%, 58.95%, 79.12%, and 33.34% in cardiac, kidney, liver, and lung allografts. The estimated percentage of host derived endothelial cells within the donor allografts was 14.04% (cardiac), 9.96% (kidney), 49.33% (liver), and 0.56% (lung). Across all patient transplant types, reverse endothelial chimerism and transplant rejection co-existed in 31.86% of patients; however, there was no significant association that could be determined between these variables (19). Hence, inferences on whether host endothelial cell integration into donor tissue is an adaptive and presumably protective phenomena or a reflection of vascular injury and rejection is unclear and yet to be determined.

## 4. Discussion

The endothelial layer serves as an interface between blood borne elements and underlying tissue, and in transplant medicine, between the self and non-self. As such it is both the site of, and an effector in immune homeostasis, and in defining the balance between rejection and tolerance (30, 31).

In HSCT, the recipient endothelium in the bone marrow niche and in the systemic vasculature may be affected by pre-existing host vulnerabilities (atherosclerosis, testosterone deficiency, heart failure) and especially by pretransplant conditioning (chemotherapy, radiotherapy, lymphodepleting regimens) that may compromise graft viability and end organ function (32, 33). Further, endothelial dysfunction is exacerbated by HLA and ABO disparity, increased synthesis of angiopoietin-2 (furthering permeability) along with loss of tight junction integrity, overexpression of adhesion molecules (ICAM, VCAM, E-selectin, P-selectin) that promote leukocyte recruitment and transmigration across the endothelium, diminished eNOS and prostacyclin that dysregulates vascular tone, and altered VEGF and FGF2. Oxidative stress, the cytokine milieu, monocyte/macrophage involvement and complement activation pathways are also implicated (34, 35). Moreover, endothelial cells act as non-professional antigen presenting cells with increased MHC class II, CD40, and ICOSL expression promoting T cell activation and chemotaxis (36).

This resultant endotheliopathy contributes to the pathogenesis of sinusoidal obstruction syndrome, engraftment syndrome, capillary leak, transplant associated thrombotic microangiopathy, graft vs. host disease and idiopathic pneumonia syndrome (34). Administration of VEGF, pigment derived endothelial factor, defibrotide, and N-acetyl-L-cysteine may ameliorate clinical outcomes (33). Animal studies published within the past decade suggest that HSCT derived donor cells contribute to the endothelial microenvironment, however the abundance of donor-derived cells varies between studies and does not address any association with GVHD or transplant rejection (10, 21). In patients that have received an ABO-incompatible HSCT, there was a statistically significant association between severe chronic pulmonary graft-vs. host disease and pulmonary endothelial chimerism (*p* < 0.001) (22), suggesting post injury seeding. Promisingly, in a mouse BMT model of acute GVHD, co-infusion of bone marrow derived EPCs mobilized to and stabilized the affected endothelium, downregulated MHC class II expression and attenuated CD3+ T cells infiltration improving pathological scores and survival outcomes in test animals (36).

In solid organ transplantation, recipient endothelial susceptibility may be exacerbated by end stage organ disease, comorbidities (hypertension, diabetes etc.), pre-existing HLA sensitization from previous blood transfusions, pregnancies, or allografts (which have been partially managed with exchange transfusions, IVIG, and depleting antibodies to attenuated B cell lineage (rituximab) or both B and T cells (thymoglobulin, alemtuzumab), as well as complicating infections. Donor derived inflammation from brain death induced cytokine storm or ischemia/reperfusion insult in the donated organ also compounds endothelial injury and activation resulting in microvascular inflammation and thrombosis. Both recipient immune cell activation as well as donor immune cells and extracellular vesicles from the transplanted organ heighten the inflammatory state that compromise graft endothelial integrity and function. Ultimately, chronic alloimmune injury leads to intimal thickening, accumulation of extracellular matrix, smooth muscle cell proliferation with resultant luminal narrowing. Here an indolent host vs. graft reaction results in transplant vasculopathy that is associated with long-term organ loss (30, 31, 37).

Some studies have investigated reverse endothelial chimerism in solid organ transplantation, both in animal models and in human studies over the last decade. Cardiac allografts demonstrated reverse endothelial chimerism at 4 weeks post-transplant in a mouse model, however quantification or association with rejection was not delineated (26). A positive association between the frequency of host-endothelial chimerism and the acute vascular rejection was seen in a rat aortic allograft model; this may be reflective of an accelerated underlying intimal injury (with associated inflammation and fibrosis) overwhelming putative stabilizing effects of a more gradual neo-endothelial seeding (27). Independent of acute vs. chronic or healing vs. progressive disease states in a rat renal transplant model, actual recipient bone marrow derived incorporated endothelium was deemed to be a rare event and endothelial regeneration from intrinsic kidney cells should also be considered at least in the syngeneic transplant model (28). A review of cardiac, kidney, liver, and lung transplants in human recipients demonstrated varying levels of reverse endothelial chimerism but no significant association with transplant rejection (19). ABO-incompatible renal transplants had decreased levels of expected blood-type antigens on graft capillaries over time suggestive endothelial chimerism but association with either graft accommodation or rejection could not be determined (10). Whereas having both a prior episode and a higher grade (II/III) of acute-T-cell mediated rejection appeared to correlate with greater number of chimeric cells per glomerular section (29). This tends to support the contention that donor graft endothelium is replaced after rejection associated vascular injury.

Whether reverse chimerism occurs primarily post graft endothelial injury or as a gradual process to “repave” the donor vasculature or a likely a combination of both is yet to be fully defined. Chimerism at the endothelial level, monocyte to endothelial transformation (particularly of VEGFR1 monocytes that express M2 phenotype to promote barrier integrity and angiogenesis) (35), cell fusion phenomena (9), and/or alloantigen incorporation by recipient antigen presenting cells may promote long term tolerance and graft survival (30); perhaps, by upregulation of protective anti-oxidant and anti-inflammatory genes (Bcl-2, Bcl-xL, HO-1) and downregulation of adhesion molecules and pro-inflammatory cytokines (35, 37, 38).

Harvesting the potential of accelerating endothelial chimerism, human placental endothelial progenitor cells are able adhere with expected alignment and morphology to decellularized vascular surfaces on rat aorta (*in vitro*) and rat kidney, lung and hindlimb (ex vivo model). Beyond the conceptual approach of promoting graft immune tolerance, placental EPCs may be harvested readily (with ABO and HLA matching), expanded, and stored for future use. Further, they retain phenotypic plasticity to adapt to the specifically seeded organ microenvironment and/or may serve as temporary vascular lining until replacement by recruited recipient endothelial cells (39). Of interest, human umbilical vein endothelial cells were co-cultured to create vessel-like structure in an *in vitro* kidney organoid model (40). Enhancing host endothelial “repaving” of the donor organ or co-infusion of donor EPCs in HSCT may prove to be promising modalities to attenuate morbidity in transplant medicine (36).

### 4.1. Limitations

There is a paucity of studies which investigated endothelial chimerism after hematopoietic stem cell or solid organ transplant and any association with graft tolerance or rejection. Given the wide variety in study designs, patient population, and outcomes analyzed, in addition to the minimal number of studies to begin with, a meta-analysis is not feasible. Many of the studies which assess sex-mismatched transplantation are lacking data on whether the donors or recipients had ever received blood transfusions, or if any of the females had miscarriages, abortions or given birth to a son—examples of a potential source of Y chromosomes and false positive signal. Additionally, cell fusion phenomena may obfuscate identification of endothelial cell ontology. Varga et al. noted endothelial chimerism within a control patient, most likely due to endothelial cell fusion in a male patient noted to have cells with double × chromosomes within tubular epithelium and double Y chromosomes within the interlobular artery (9). Further, tissue specific or circulating mesenchymal precursor cells may confound clear identification of endothelial chimerism and preclude inferences on clinical significance. Heterogeneity of techniques to assess chimerism and dependence on a single endothelial cell surface marker pose challenges to study design and conclusions. Moreover, functional assessment of presumed chimeric endothelial cells is challenging (and lacking at the cellular level) also obscuring clinical implications.

### 4.2. Future directions

Given the level of controversial data regarding the frequency of which endothelial chimerism occurs after a bone marrow or solid organ transplant, a broad multimodal study covering thousands of patients *via* coordination between multiple sites (including harvesting data from already existing biopsy samples correlated to clinical outcomes) may be necessary to determine baseline endothelial chimerism levels and to validate or refute current data and associated clinical implications.

One aspect of this chimerism suggests that immune-mediated endothelial cell injury either in a transplanted organ due to ABO incompatibility or GVHD after a HSCT activates a repair response leading to bone marrow or organ derived endothelial cells to migrate to this point of possible de-endothelialization. Monitoring the number and activation state of circulating endothelial cell populations in the transplant setting would allow an assessment of their genotypic ontology and phenotypic expression. Human endothelial progenitor cells (EPCs) express a variety of cell surface markers similar to those expressed by vascular endothelial cells, adhere to endothelium at sites of injury, have expansive potential and are purported to assist in vascular intimal healing. Circulating endothelial cells (CEC) represent peripheral blood cell subpopulation detached from an established vascular network characterized by mature endothelial features with limited proliferative potential. Flow cytometry can identify and quantify these cell subtypes allowing for inferences over time in cheremic incidence and associated disease state (41). Further an assessment of angiogenic factors such as VEGF, FGF, angiopoietin -1 and -2, Tie-2, thrombospondin-1, heparan sulfate proteoglycans, etc especially in hypoxic microenvironments such as organ rejection may serve as modulating factors for this chimerism. The injury to the vessel may signal procoagulant factors such as von Willebrand's factor, tissue factor, EPCR, D-dimer and thrombin-antithrombin complexes and complement activation. This analysis may be complementing by assessing shed endothelium microparticles (which have transmembrane proteins and surface markers present on their phospholipid bilayer and contain cytosolic components such as enzymes, transcription factors and mRNA from their parent cells) (42). Pairing blood sampling with pathology from needle biopsy or even whole explanted donated organs (the latter in the event of graft failure or at autopsy) may yield further mechanistic insights.

To answer these questions, use of spatial transcriptomics whereby quantification of mRNA (as a proxy for gene expression) in relation to the spatial context of cells within tissue architecture may be sought. Especially relevant here would be the zone of the anastomosis between the donor organ vessels and the recipient's arterial and venous vasculature. The goal being broad transcriptome profiling and high gene detection efficiency at the single cell resolution level to infer cell ontology and functional state (even at the proteome level). Single-molecule fluorescent *in situ* hybridization in series and sequentially to create combinatorial barcoding to reconstruct gene expression in 3D and cross referenced to tissue atlases (currently primarily focused on brain, lung and breast tissue in humans) would likely yield such information. Optimizing signal to noise ratio, limiting optical crowding, balancing spatial resolution with tissue field of view, leveraging automation for high throughput analysis, sharing open-source code, integrating data bases, moving beyond institute of origin specific protocols to commercial systems with decreasing cost would all yield beneficial insights (43). As an example, single cell transcriptome methods have been successfully applied to define the heterogeneity and chimerism of endothelial cells in a mouse liver cancer model (44).

Incorporation of above cited detection techniques (45) would be required to identify previously undiagnosed chimeric states and aid in the understanding of pathophysiology and clinical management.
